# Medical prospects of cryptosporidiosis *in vivo* control using biofabricated nanoparticles loaded with *Cinnamomum camphora* extracts by *Ulva fasciata*

**DOI:** 10.14202/vetworld.2024.108-124

**Published:** 2024-01-18

**Authors:** Nesreen Allam Tantawy Allam, Ragaa Abd El-Fatah Hamouda, Doaa Sedky, Mahinour Ezzeldin Abdelsalam, Mona Ebrahim Hussien Abd El-Gawad, Noha Mahmoud Fahmy Hassan, Dina Aboelsoued, Amal M. Abou Elmaaty, Muhammad A. Ibrahim, Hanan Anwar Aly Taie, Ashraf Samir Hakim, Hassan Mohamed Desouky, Kadria Nasr Abdel Megeed, Marwa Salah Abdel-Hamid

**Affiliations:** 1 Department of Parasitology and Animal Diseases, Veterinary Research Institute, National Research Centre, 33 El Buhouth Street, Dokki, P.O. Box: 12622, Giza, Cairo, Egypt; 2 Department of Microbial Biotechnology, Genetic Engineering and Biotechnology Research Institute, University of Sadat City, 5^th^ Zone, Sadat City, Munofia, Egypt; 3 Department of General Biology, Center of Basic Sciences, Misr University for Science and Technology, Al Motamayez District, 6^th^ of October, Giza, Cairo, Egypt; 4 Cytogenetics and Animal Cell Culture Lab., National Gene Bank, Agriculture Research Center, 9 Gamaa Street, Giza, Cairo, Egypt; 5 Department of Animal Reproduction and Artificial Insemination, Veterinary Research Institute, National Research Centre, 33 El Buhouth Street, Dokki, P.O. Box: 12622, Giza, Cairo, Egypt; 6 Department of Plant Biochemistry, Agriculture and Biological Researches Institute, National Research Centre, 33 El-Bohouth St. (Former El-Tahrir St.), Dokki, P.O. 12622, Giza, Cairo, Egypt; 7 Department of Microbiology and Immunology, Veterinary Research Institute, National Research Centre, 33 El Buhouth Street, Dokki, P.O. Box: 12622, Giza, Cairo, Egypt

**Keywords:** blood biomarkers, *Cinnamomum camphora*, *Cryptosporidium parvum*, cytokines, Egypt, genotoxicity, green nanoparticles, rats, *Ulva fasciata*

## Abstract

**Background and Aim:**

Global efforts are continuing to develop preparations against cryptosporidiosis. This study aimed to investigate the efficacy of biosynthesized *Ulva fasciata* loading *Cinnamomum camphora* oil extract on new zinc oxide nanoparticles (ZnONPs shorten to ZnNPs) and silver nanoparticles (AgNPs) as alternative treatments for *Cryptosporidium parvum* experimental infection in rats.

**Materials and Methods:**

Oil extract was characterized by gas chromatography-mass spectrometry, loaded by *U. fasciata* on ionic-based ZnO and NPs, and then characterized by transmission electron microscopy, scanning electron microscopy, and X-ray diffraction. Biosafety and toxicity were investigated by skin tests. A total of 10^5^ *C. parvum* oocysts/rat were used (n = 81, 2–3 W, 80–120 g, 9 male rats/group). Oocysts shedding was counted for 21 d. Doses of each preparation in addition to reference drug were administered daily for 7 d, starting on post-infection (PI) day (3). Nitazoxanide (100 mg) was used as the reference drug. After 3 weeks, the rats were sacrificed for postmortem examination and histopathological examination. Two blood samples/rat/group were collected on the 21^st^ day. Ethylenediaminetetraacetic acid blood samples were also used for analysis of biochemistry, hematology, immunology, micronucleus prevalence, and chromosomal abnormalities.

**Results:**

*C. camphora* leaves yielded 28.5 ± 0.3 g/kg oil and 20 phycocompounds were identified. Spherical and rod-shaped particles were detected at 10.47–30.98 nm and 18.83–38.39 nm, respectively. ZnNPs showed the earliest anti-cryptosporidiosis effect during 7–17 d PI. Other hematological, biochemical, immunological, histological, and genotoxicity parameters were significantly fruitful; hence, normalized pathological changes induced by infestation were observed in the NPs treatments groups against the infestation-free and Nitazoxanide treated group.

**Conclusion:**

*C. camphora*, *U. fasciata*, ZnNPs, and AgNPs have refluxed the pathological effects of infection as well as positively improved host physiological condition by its anticryptosporidial immunostimulant regenerative effects with sufficient ecofriendly properties to be proposed as an alternative to traditional drugs, especially in individuals with medical reactions against chemical commercial drugs.

## Introduction

*Cryptosporidium*, a single-celled parasite, has broad host specificity [1]. *Cryptosporidium parvum* is the most common infectious species, particularly in neonates and/or young ruminants [2, 3]. Cryptosporidiosis is more common in young animals. Severity depends on many factors, including age, host immunity, infectious dose, geographical distribution, season, and mixed infection with other pathogens [4]. Infected individuals experience profuse watery diarrhea associated with many complications such as exsiccosis, electrolyte loss, and metabolic acidosis [4]. Severe symptoms include inappetence, weight loss, lethargy, dehydration, and death [5, 6]. In addition to its high morbidity rate, *C. parvum* is of significant concern to farmers and veterinary authorities because mortality data indicate that *C. parvum* mostly infects calves aged 1 month or less, whereas adults harbor symptomless infection [2, 3]. Control of cryptosporidiosis remains a global challenge in veterinary medicine [7–9]. Therefore, there is a need for continued efforts to interrupt oocyst transmission through the ingestion of contaminated water and food and/or contact with infected animals [9]. Although several preparations have been tested, no effective treatment or vaccine against cryptosporidiosis has been developed [8–10]. Nitazoxanide (NTZ) has been approved by the United States Food and Drug Administration for the treatment of human cryptosporidiosis, particularly in children and immunocompetent individuals [11]. Halofuginone lactate has been licensed for veterinary use in Europe; however, it is less effective once diarrhea is established [12]. However, the limitations of medications for the treatment and/or control of cryptosporidiosis, such as mefloquine [13], S-methylcysteine [14], NTZ in patients with hepatic conditions [11], and halofuginone lactate in individuals with diarrhea [12], triggered trials using nanoparticles (NPs) simultaneously to ameliorate the adverse effects of parasites on the host intestine [11]. Some plant extracts, such as cinnamon and onion [15], garlic, curcumin, black seed [16], moringa, pomegranate [2], ginger, ginseng, and sage [3], have also been tested against *Cryptosporidium* spp. In addition, *Saccharomyces cerevisiae* fermentation products have been utilized as a natural alternative for the control of bovine cryptosporidiosis. Propolis (bee glue) extracts also have anticryptosporidial activity [16].

Nanoparticle as a therapy, which provide a general strategy that might help improve the efficacy of any type of drug targeting *Cryptosporidium* parasites and offer anticryptosporidial activities [16]. Nanoparticles display unusual properties related to their size, shape, and morphology that allow them to interact with animals, plants, and microorganisms [17]. Hence, they have gained distinction in technological developments due to their unique physical, chemical, and biological properties, with improved performance compared to their bulk materials. Various types of NPs have presented alternative methods for controlling infectious causes and enhancing body performance in livestock. Carbon, organic, inorganic, and composite nanomaterials are classified [18, 19]. They include different metals such as silver NPs (AgNPs) and metal-oxide nanomaterials such as zinc oxide NPs (ZnONPs shorten to ZnNPs). Moreover, they are non-toxic, biocompatible, and stable compared with organic nanomaterials. Silver NPs are the most fascinating among many other inorganic NPs that have been used in biomedical appliances, playing a key role in nanoscience and nanomedicine. Silver NPs have also been used to treat cryptosporidiosis [20, 21]. They are assumed to break the walls of *C. parvum* oocysts. In contrast, ZnNPs possess a large surface area, display greater toxicity, and exhibit anticancer activities [20–22]. ZnNPs have been found to kill *Giardia intestinalis* in experimentally infected mice, decrease the incidence of apoptosis, protect intestinal cells, and aid in their regeneration. In addition, they possess antibacterial activity as they hinder biofilm formation by *Streptococcus mutans*. Both ZnNPs and AgNPs showed effective antiparasitic pharmacokinetics against *Meloidogyne incognita* nematodes [20–22]. *U. fasciata*, a marine alga, produces a variety of primary and secondary metabolites, including polysaccharides, sterols, minerals, proteins, vitamins, fatty acids, lipids, carbohydrates, and potential therapeutic compounds [23, 24]. Therefore, *U. fasciata* is seriously considered for the development of novel drugs and/or formulas for multipartite structures such as NPs in the pharmaceutical sector [25]. *U. fasciata* has been reported to improve the stability, homogeneity, and presentation of loaded green NPs. In addition, they improve the nutritive value as well as the antigenicity of biofabricated particles when considering their nano size [7, 26, 27].

Essential oil-based antiparasitics have a wide range of biomedical applications, especially in the veterinary field, with several advantages such as being readily available, renewable, and readily degraded to minimize the side effects [7]. *Cinnamomum camphora* oil possesses anticoccidial, antiviral, antimicrobial, insecticidal, anticancer, and oocysticidal biological properties *in vitro* [28]. *In vivo* antidiarrheic effects have also been reported [28, 29].

The present study aimed to conjugate *C. camphora* oil extract with both ZnNPs and AgNPs mediated by *U. fasciata alga* collected from the shores of the Mediterranean Sea at Abo-Quier beach, Alexandria, Egypt, then to investigate their potential impact as therapeutic and medical agents against cryptosporidiosis *in vitro* and *in vivo* in rats.

## Materials and Methods

### Ethical approval

All experimental procedures were performed in accordance with the ethical guidelines of scientific committees in both the National Research Centre (Approval No. NRC-16231) and the Genetic Engineering and Biotechnology Research Institute, University of Sadat City (Approval No. IACUC-GEBRI-USC-20-2020).

### Study period and location

The study was performed from May 2019 to May 2022 at National Research Centre, Genetic Engineering and Biotechnology Research Institute, and National Gene Bank.

### Biofabrication of NPs

#### Oil extraction and characterization

Briefly, 1 Kg of *C. camphora* leaves were hydro-distilled for over 4 h using a modified Clevenger apparatus according to a previous study. The extracted essential oil volume was determined and recorded based on weight. The oil was then dehydrated over anhydrous sodium sulfate and stored in dark glass vials in a freezer until used for gas chromatography-mass spectrometry (GC/MS) and biological analysis [30].

The oil’s physicochemical properties were determined according to Egyptian standards equivalent to those of the International Standard Organization. The chemical composition of the oil was studied by (GC/MS; Agilent 7890 B and 5977A, USA) [30]. We identified the components by comparing the mass spectral fragmentation patterns with those found in databases [31].

#### Biosynthesis of NPs

*Alga* was collected from the shores of the Mediterranean Sea at Abo-Quier Beach in Alexandria, Egypt [29]. The sample was then washed, dried, and ground. Dry *U. fasciata* (1 g) in 100 mL double-distilled water was boiled for 1 h to obtain the algal extract, which was then filtered. Next, 10 mL of extract was added to 40 mL of double-distilled water containing 0.02 M Zn acetate and dehydrated by constant stirring for 10 min. Then, 2.0 M NaOH was added and the mixture was stirred for another 2 h. A pale-white precipitate was obtained, which was then filtered, washed 2 times with distilled water, 1 time with absolute ethanol, and dried overnight at 60°C [32].

Biosynthesis of AgNPs was achieved by adding 0.017 g of AgNO_3_ to 90 mL of double-distilled water and stirring, after which 10 mL of *U. fasciata* extract was added drop-wise, and the mixture was left on a stirrer until the color changed to pale brown [29]. Thereafter, 10 mL of *C. camphora* oil was mixed with 90 mL of both AgNPs and ZnNPs with thorough stirring for 10 min at 200 rpm and then stored at 4°C [29]. Transmission electron microscopy (TEM) and scanning electron microscopy (SEM) were used to determine the morphology and particle size of the prepared NPs. The chemical structures of green-synthesized AgNPs and ZnNPs were determined using energy-dispersive X-ray spectroscopy (EDX).

#### Biosafety of all preparations

Ten rats divided into five groups were inoculated subcutaneously with 0.5 mL of each new preparation and then kept under observation for 7 days to evaluate toxicity effects, including body weight loss, systemic effects, and behavioral variations (posture, locomotion, awareness of surroundings, reaction to stimulus, and stress indicators as barbering and diarrhea) [33]. Sensitization of the immune system was estimated through a skin test, wherein 0.2 mL of each preparation and physiological solution was inoculated intradermally [33]. Skin reactions were measured at 24 and 48 h post-infection (PI). Erythema and/or edema with 2–5 mm diameter at the point of injection were considered positive signs.

### Parasite

*Cryptosporidium parvum* isolate (GenBank: ON730708) previously identified by Aboelsoued *et al*. [34] using the *Cryptosporidium* oocyst wall protein gene by polymerase chain reaction. Fecal samples were collected from buffalo calves (aged 10–20 days) reared by local farmers in the Beni-Suef Governorate, Egypt. Before experimental infection, oocysts were concentrated and counted using a hemocytometer in phosphate-buffered saline solution [14, 23].

### Experimental infection

#### Animals

Male rats (n = 81), 2–3 weeks old and weighing 120 g, were housed in well-ventilated cages with perforated covers in the NRC Animal House, Egypt. They were supplied with standard pellets and had free access to food and water. The rats were euthanized rapidly and painlessly at the end of the experiment. After post-mortem examination, small intestine parts were collected, followed by hygienic disposal of the carcasses.

#### Infection and treatments

The male rats were divided into nine groups (9 rats/group) (Table-1). The experiment involved infection with 10^5^ *C. parvum* oocysts (a single dose in gastric tubes 1 h before meal) [3, 33, 34]. Therapeutic doses of *C. camphora* oil pure extract, AgNPs (50%), and ZnNPs (50%) were 20 μL/kg body weight according to LCD_50_ and LCD_90_ calculated during *in vitro* oil and nano preparation characterization. Doses were administered daily using gastric tubes 1 h before meals for 7 consecutive days. Therapeutic doses were started on the 3^rd^ d PI. All animals were sacrificed after 3 weeks.

**Table-1:** Rats Groups designation during the experimental infestation and treatment study.

Groups	Description	Treatments
G1	Healthy, non-infected, and non-treated rats	Control negative for infection and treatments
G2	Experimentally infected with 10^5^ *C. parvum* oocysts	Control positive for infection but non-treated
G3	Healthy, non-infected rats, treated with *C. camphora* oil	Control for oil extract treatment
G4	Healthy, non-infected rats, treated with AgNPs (50%)	Control for AgNPs treatment
G5	Healthy, non-infected rats, treated with ZnNPs (50%)	Control for ZnNPs treatment
G6	*C. parvum*-infected rats treated with NTZ Reference drug	Cryptonaz^®^ (100 mg NTZ, Copad Pharma, Egypt) treatment
G7	*C. parvum*-infected treated with extract	*C. camphora* oil treatment
G8	*C. parvum*-infected treated with AgNPs	AgNPs 50% treatment
G9	*C. parvum*-infected rats treated with ZnNPs	ZnNPs 50% treatment

*C. parvum*=*Cryptosporidium parvum, C. camphora*=*Cinnamomum camphora*, AgNPs=Silver nanoparticles, ZnNPs=Zinc oxide nanoparticles, NTZ=Nitazoxanide

#### Shedding of C. parvum oocysts

Rats’ fecal samples were collected daily from the third day PI till the end of the experiment (21 d). Then samples were examined using MZN staining technique under microscope (Olympus Corporation CX41, Japan) for determination of the number of oocysts output counted for each group in 50 fields (oil immersion × 1000).

#### Hematological and metabolic profiles

Two blood samples from each rat/group were collected; one in ethylenediaminetetraacetic acid tubes for hematological studies and the other in plain tubes, and clotting was performed to obtain serum for the measurement of biochemical parameters and immunological markers. Sampling was conducted from day zero to day 21 PI. Serum concentrations of total proteins (biuret method), albumin, globulin, albumin-globulin ratio, and cholesterol were determined using kits (Linear Chemicals; Barcelona, Spain) [35]. Liver enzymes such as alanine aminotransferase (ALT), aspartate aminotransferase (AST), serum urea, and serum urea nitrogen were determined using kits **(**Linear Chemicals) [36].

#### Pro-inflammatory and oxidative stress biomarkers

Interferon-gamma (IFN-γ) and interleukin (IL)-4 levels were analyzed using commercially available kits (Sulong Biotech Co., China). The sandwich enzyme-linked immunosorbent assay detection range was determined by the manufacturer to be 2.6–160 pg/mL and 1–80 pg/mL for IFN-γ and IL-4, respectively (Sulong Biotech Co.). The enzymatic activities of glutathione peroxidase (GPx), superoxide dismutase (SOD), and catalase (CAT) were determined [37] at A340/min over a 3-min period, A560 for 5 min, and A520 after 1 min, respectively, at 25°C against blanks. The readings represent U/mL of the enzyme (Biodiagnostics, Giza, Egypt).

#### Histopathological studies

All healthy and infected rats were sacrificed at the day 21 PI (the end of experiment). Ilea of all rats were fixed directly in 10% formalin for 24 h, dehydrated, cleared, embedded in paraffin, sectioned at 4 μm then stained with H&E staining. Slides examination microscopically was carried out for comparative pathological evaluation of infection course and therapeutic effects of applied preparations in association to reference drug used [38].

#### Genotoxicity assessment

Micronucleus frequency

We estimated the prevalence of micronuclei as described previously by Essa *et al*. [39]. Bone marrow was retrieved from the femur by flushing with 1 mL fetal calf serum (Sigma-Aldrich S.r.l.-Mailand, Italy) and centrifuged at 265× *g* for 15 min. The cell suspensions were decanted and fixed in a cold 3:1 methanol: acetic acid solution. Two smears per rat were formed by dropping pellets on slides at a 45° angle, drying for 20 min, and staining with Giemsa stain [39]. Micronucleated polychromatic erythrocytes were counted under a light microscope (Leica DM2500, England) at 100 magnification using immersion oil.

Chromosomal aberrations percent

Each rat was intraperitoneally injected with colchicine (3 mg/kg body weight) 2 h before sacrifice. Cervical dislocation and femoral bone resection were performed in normal saline. The epiphyses were severed, and bone marrow was aspirated with 2.2% sodium citrate solution (w/v). Subsequently, the suspension was centrifuged at 537× *g* for 10 min, decanted, and replaced with 0.075 M potassium chloride for 30 min; the same steps were repeated. Each supernatant was decanted and replaced with a freshly prepared cold fixative (3:1 v/v methanol: glacial acetic acid), which was allowed to stand for 10 min before centrifugation for 10 min at 537× *g*. The fixation and centrifugation process were repeated 3 times. Fixed cells were dropped from 30 to 40 cm height onto clean, dry, grease-free slides, and air-dried for 10 min before being stained with 5% Giemsa (v/v, stock Giemsa stain/distilled water) [39]. The slides were screened for chromosomal abnormalities at 1000 magnification. Fifty well-spread metaphases were scored per rat, and the mitotic index of approximately 3000 cells/concentration was examined; significance was estimated by student’s t-test (p < 0.05).

### Statistical analysis

Collected data are presented as mean ± SEM (Standard error of the mean). Simple one-way analysis of variance was used to study the effect of treatment on studied parameters. Duncan’s multiple range test was used to differentiate between significant means at p *<* 0.05 [40] using SPSS software version 20.0. for Windows (SPSS Inc., Chicago IL USA).

## Results

### Gas chromatography-mass spectrometry analysis of oil

Leaf yield was 28.5 ± 0.3 g/kg. In the GC-MS analysis, 20 phycocompounds were identified based on retention time, % peak area, molecular formula, and weight. These components include monoterpenes, sesquiterpenes, and oxyterpenes. The main components were low-polarity volatile acids, suggesting that the leaves are a rich source. Eleven monoterpenes, five sesquiterpenes, and four oxyterpenes were identified in the extracts. Oxyterpenes were the major compounds; d-borneol (66.9%), 1,8-cineole (4.2%), camphor (0.9%), and α-terpineol (0.5%), together representing 72% of the extract. The rest of the compounds were monoterpenes (24.39%), mainly α-pinene (8.32%), limonene (4.58%), camphene (4.25%), β-pinene (2.19%), β-myrcene (1.98%); and sesquiterpenes (3.71%), mainly trans-caryophyllene (2.01%), α-humulene (1.21%), and γ-elemene (0.41%). Chemical characterization of the solubility in ethanol, refractive index, relative density, and optical rotation was performed (data not shown), and the results were in agreement with previous reports on *C. camphora* leaf oil extract.

### Physicochemical characters of biofabricated NPs

#### Electron microscopy

**Figure-1:**
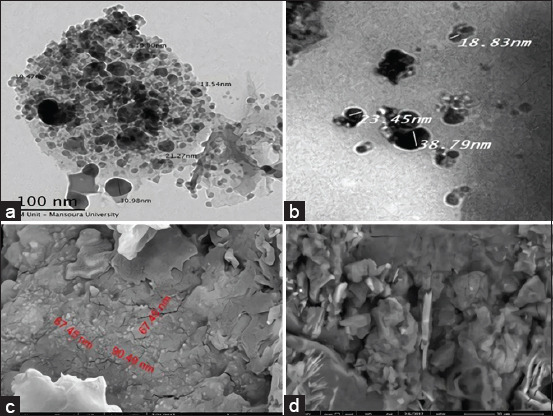
Images of *Cinnamomum camphora*-*Ulva fasciata-*AgNPs and *C. camphora -Ulva fasciata*-ZnNPs Characterization. (a and b) TEM images (c and d) SEM images. AgNPs=Silver nanoparticles, ZnNPs =Zinc oxide nanoparticles, TEM=Transmission electron microscopy, SEM=Scanning electron microscopy.

Transmission electron microscopy images of AgNPs and ZnNPs are shown in Figures-1a and b, respectively. The AgNPs were spherical in shape and 10.47–30.98 nm in size, whereas the ZnNPs were oval in shape and 18.83–38.39 nm in size; both were well dispersed. The morphologies of AgNPs and ZnNPs were observed through SEM (Figures-1c and d). ZnNPs had curled surface rod flakes, while AgNPs were spherical with a narrow size distribution through the flakes.

#### X-ray diffraction (XRD) analysis

**Figure-2:**
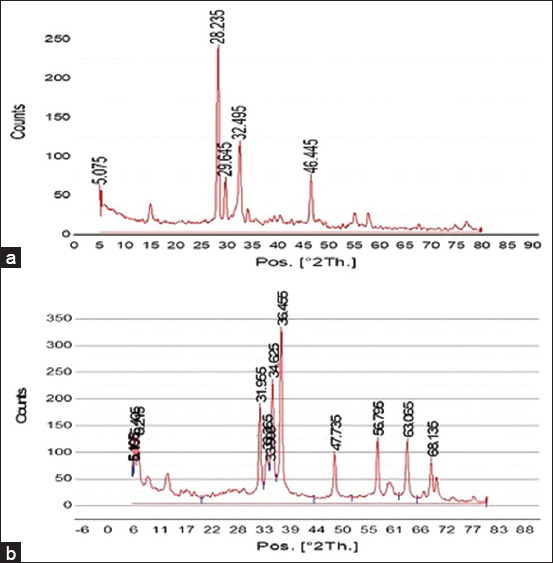
(a) X-ray diffraction pattern showing the crystallographic structure, chemical composition, and physical properties of *Cinnamomum camphora*-*Ulva fasciata-*silver nanoparticles and (b) *Cinnamomum camphora*-*Ulva fasciata*-zinc oxide nanoparticles.

X-ray diffraction was used to investigate the crystallite phases of both AgNPs and ZnNPs (Figures-2a and b). Peaks of AgNPs were observed at 2 theta 5.075°, 28.23°, 29.64°, 32.49°, and 46.44°, respectively. Values of 2 theta were 5.1°, 5.45°, 31.95°, 33.09°, 33.66°, 34.62°, 36.54°, 47.73°, 56.79°, 63.06°, and 68.13° for ZnNPs. These sharp peaks indicated the crystallinity of both AgNPs and ZnNPs.

#### Biosafety and toxicity of the preparations

No toxic effects were observed in rats during the inspection period (7 days) due to medical treatment with the preparations. Body weight, physiological systemic reflexes, and behavioral profiles were all within the normal range (data not shown). Immune-dependent cutaneous reactions revealed significant increases in cellular infiltration of natural killer cells, lymphocytes, and macrophages, accompanied by pro-inflammatory cytokine production as well as chemotaxis (Table-2).

**Table-2:** Cellular infiltrations determined by skin thickness test in experimentally infected rats treated with different preparations applied during the study.

Rats Groups	21-day Post-treatment		90-day Post-treatment (cumulative effect)	
	
	after 24 h	after 48 h	after 24 h	after 48 h
G1: Healthy, non-infected and non-treated	0.4 ± 0.240	1.0 ± 0.450	0.4 ± 0.240	1.0 ± 0.450
G3: Healthy, non-infected, treated with *C. camphora* oil	0.0 ± 0.000	1.2 ± 0.730	0.0 ± 0.000	2.4 ± 0.600*
G6: *C. parvum*-infected rats treated with NTZ Reference drug	0.2 ± 0.200	1.0 ± 0.320	0.2 ± 0.200	1.0 ± 0.320
G7: *C. parvum*-infected treated with extract	0.2 ± 0.920**	4.2 ± 0.490*	2.2 ± 0.920**	4.6 ± 0.680*
G8: *C. parvum*-infected treated with AgNPs	2.0 ± 0.000**	2 ± 0.000**	0.4 ± 0.245	0.8 ± 0.449
G9: *C. parvum*-infected rats treated with ZnNPs	3.2 ± 0.490*	4.2 ± 0.490*	1.8 ± 0.735**	4.6 ± 0.245*

(*) considered significance at p *<* 0.001, while (**) considered significance at p *<* 0.05, *C. parvum*=*Cryptosporidium parvum, C. camphora*=*Cinnamomum camphora*, AgNPs=Silver nanoparticles, ZnNPs=Zinc oxide nanoparticles

### Medical prospects of preparations in experimentally infected rats with *C. parvum*

#### Oocysts’ shedding in treated rats

A fecal smear examination was performed to evaluate *C. parvum* oocyst shedding in experimentally infected rats in response to the treatment. Gradual reduction in oocyst shedding in the positive control group from day 11 to day 21 PI continued until almost no oocysts were found (Table-3). All the infected rat groups shed oocysts on day 3 PI, which diminished on days 9 and 11 in the *C. camphora* oil-and NTZ-treated groups, respectively, and on day 7 in both the ZnNPs- and AgNPs- treated rats’ groups. A statistically significant reduction (p > 0.01) in oocyst shedding was observed in the four treated rat groups compared with that in the infected non-treated rat group. Negligible counts of oocysts or no oocysts were observed on days 21, 21, 17, and 20 PI for *C. camphora* oil-, NTZ-, ZnNPs-, and AgNPs -treated rats, respectively. ZnNPs showed a better effect than AgNPs on *C. parvum* oocyst count in infected rats, where no oocysts were found on days 17 and 18 PI, with no statistically significant differences (p > 0.01).

**Table-3:** Therapeutic effect of different preparations applied during the study on *Cryptosporidium parvum* oocysts fecal shedding in experimentally infected rats.

Days PI	G2: Experimentally infected with 105 *C. parvum* oocysts	G6: *C. parvum*- infected rats treated with NTZ reference drug	G7: *C. parvum*- infected treated with extract.	G8: *C. parvum*- infected treated with AgNPs	G9: *C. parvum*- infected rats treated with ZnNPs	p-value
Day 3	82.4 ± 2.74^ab^	84.20 ± 1.90^a^	83.03 ± 1.10^b^	83.20 ± 1.40^b^	82.10 ± 1.20^ab^	NS
Day 4	84.4 ± 1.05^a^	84.80 ± 1.60^a^	83.00 ± 1.20^ab^	83.23 ± 1.50^ab^	82.02 ± 1.10^b^	NS
Day 5	90.6 ± 1.34^a^	88.00 ± 1.58^ab^	85.60 ± 1.34^c^	86.40 ± 1.30^bc^	86.00 ± 1.41^c^	<0.05
Day 6	94.8 ± 0.84^a^	93.60 ± 1.14^b^	87.60 ± 1.14^c^	93.80 ± 1.30^b^	81.00 ± 1.00^d^	<0.01
Day 7	96.2 ± 1.30^ab^	96.60 ± 1.14^ab^	88.40 ± 1.14^c^	83.80 ± 0.84^d^	70.60 ± 1.95^e^	<0.01
Day 8	99.2 ± 0.84^a^	97.02 ± 1.02^a^	89.80 ± 0.84^b^	72.60 ± 2.03^c^	55.24 ± 1.10^d^	<0.01
Day 9	100.6 ± 1.50^a^	99.40 ± 0.89^a^	73.40 ± 1.67^b^	71.80 ± 3.63^c^	24.60 ± 1.14^d^	<0.01
Day 10	106.8 ± 1.10^a^	101.80 ± 2.17^a^	65.00 ± 1.58^c^	53.00 ± 2.12^b^	20.20 ± 1.30^d^	<0.01
Day 11	90.6 ± 1.52^a^	90.26 ± 1.20^a^	44.40 ± 1.14^b^	30.40 ± 6.43^d^	13.20 ± 1.30^c^	<0.01
Day 12	84.0 ± 2.12^a^	89.80 ± 0.84^a^	26.80 ± 1.3^b^	14.60 ± 1.14^d^	8.20 ± 1.30^e^	<0.01
Day 13	58.4 ± 1.82^a^	53.40 ± 1.82^a^	23.00 ± 1.87^b^	7.60 ± 0.55^c^	6.00 ± 0.71^c^	<0.01
Day 14	43.0 ± 2.30^a^	32.00 ± 1.87^b^	20.40 ± 1.14^b^	6.00 ± 0.71^c^	3.00 ± 1.00^d^	<0.01
Day 15	34.0 ± 1.70^b^	17.06 ± 1.30^b^	19.10 ± 1.10^b^	3.10 ± 1.20^c^	2.50 ± 0.50^c^	<0.01
Day 16	18.6 ± 1.34^a^	12.40 ± 0.90^c^	13.04 ± 0.20^b^	2.30 ± 0.30^d^	0.38 ± 0.20^e^	<0.01
Day 17	15.4 ± 2.52^a^	12.20 ± 1.64^c^	13.80 ± 0.70^b^	1.60 ± 0.55^d^	0.00 ± 0.00^e^	<0.001
Day 18	13.2 ± 1.87^a^	5.20 ± 0.04^c^	8.22 ± 1.50^b^	0.80 ± 0.40^c^	0.00 ± 0.00^c^	<0.01
Day 19	5.2 ± 0.80^a^	3.28 ± 0.91^bc^	2.20 ± 0.40^bc^	0.40 ± 0.03^d^	0.00 ± 0.00^d^	<0.01
Day 20	0.5 ± 0.14^ab^	0.40 ± 0.80^ab^	0.40 ± 0.14^ab^	0.00 ± 0.00^c^	0.00 ± 0.00^c^	<0.01
Day 21	0.1 ± 0.01^a^	0.00 ± 0.00^b^	0.00 ± 0.00^b^	0.00 ± 0.00^b^	0.00 ± 0.00^b^	NS

Data expressed as means ± standard deviation. Means followed by different superscripts within row (a, b, c, d, and e) are significant at p *<* 0.05. NS=Non-Significant, *C. parvum*=Cryptosporidium parvum, AgNPs=Silver nanoparticles, ZnNPs=Zinc oxide nanoparticles, NTZ=Nitazoxanide

#### Hematological profiles

**Figure-3:**
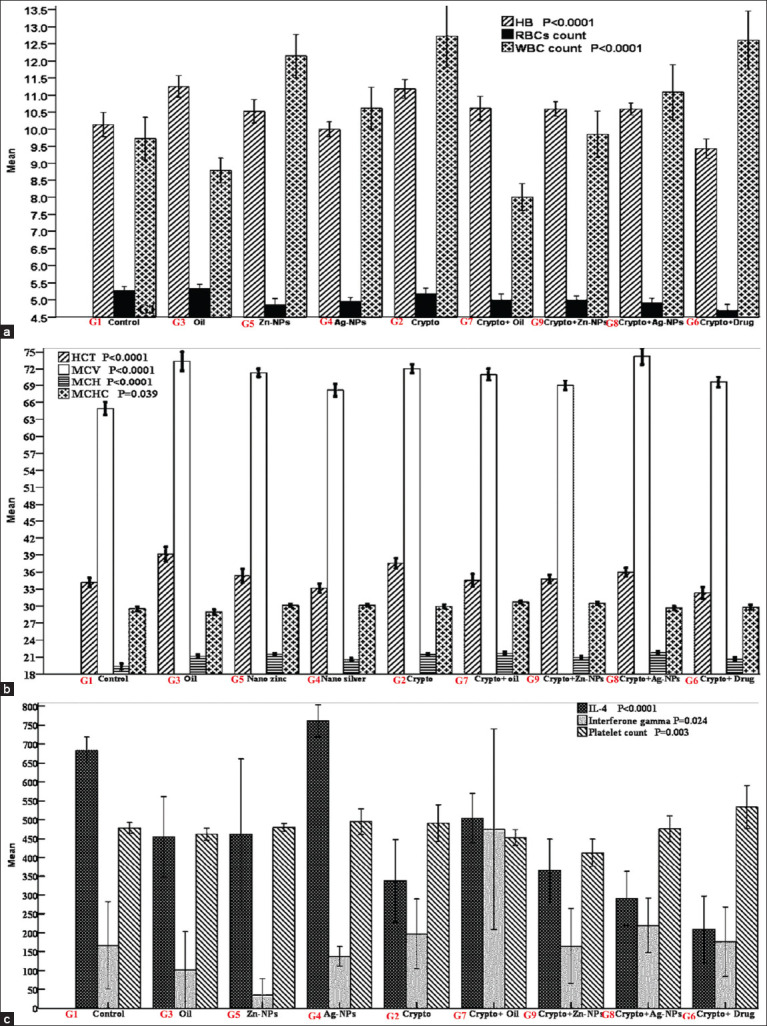
Hematological and metabolic analysis results of studied rat groups showing: (a) Mean HB, RBCs, and WBCs; (b) Mean HCT, MCV, MCH, and MCHC; and (c) Mean Blood Platelets, IFN-γ, and IL-4 values in treated rats groups with error bars where G1 (Control); G2 (Crypto); G3 (Oil); G4 (AgNPs); G5 (ZnNPs); G6 (Crypto-Drug); G7 (Crypto+Oil); G8 (Crypto+Ag-NPs); and G9 (Crypto+Zn-NPs). HB: Hemoglobin, RBCs: Red blood cells, WBCs: White blood cells, HCT: Hematocrit, MCV: Mean corpuscular volume, MCH: Mean corpuscular hemoglobin, and MCHC: ???

Hemoglobin (HB) levels were low in the G6 (9.43 ± 0.28), G1 (10.14 ± 0.35), and G4 (9.94 ± 0.26) groups (p < 0.0001). G3 (11.17 ± 0.29) and G2 (11.14 ± 0.28) rats had the highest HB levels (Figure-3a). Groups G5 (10.58 ± 0.30), G9 (10.59 ± 0.28), G7 (10.62 ± 0.36), and G4 (10.59 ± 0.18) reported insignificantly higher levels, whereas G1, G6, and G4 had insignificantly lower levels compared to those in the G7 group (Figure-3a). Red blood cells counts were not affected by the treatments, although the G6 group showed the lowest count (4.70 ± 0.17) compared to the G3 (5.23 ± 0.14) and G1 (5.27 ± 0.12) groups (Figure-3a). Both G6 (12.61 ± 0.85) and G2 (12.95 ± 0.94) groups had the highest white blood cells (WBC) count (p = 0.0001), whereas G7 (8.00 ± 0.39), G3 (8.80 ± 0.35), G1 (9.73 ± 0.62), and G9 (9.85 ± 0.68) groups had lower WBC counts with no significant differences among them (Figure-3a). G4 (11.00 ± 0.67), G8.

The values of Hematocrit (HCT) (Figure-3b) were significantly low (p < 0.0001) in G6 (32.30 ± 0.33) and G4 (32.83 ± 0.91) groups. G1 (34.14 ± 0.81), G7 (34.54 ± 1.12), and G9 (34.78 ± 0.74) groups showed insignificantly higher values (Figure-3b). The values of HCT increased insignificantly in G5 (35.63 ± 0.81) and G8 (35.95 ± 0.78) groups. Both G3 (37.43 ± 0.74) and G2 (38.67 ± 1.25) groups showed the highest values of HCT (Figure-3b). The lowest values of Mean corpuscular volume (MCV) (p < 0.0001) could be seen in the G1 group (64.91 ± 1.17) (Figure-3b). Significant increases in the MCV values were observed in the G4 (68.33 ± 0.33), G9 (69.04 ± 0.85), G6 (69.61 ± 0.99), G5 (70.63 ± 0.86), and G7 (71.00 ± 1.07) groups. G3 (73.47 ± 1.56), G2 (72.10 ± 0.79), and G8 (74.23 ± 1.47) groups had the highest values (Figure-3b). The lowest values of mean corpuscular hemoglobin (MCH) (p < 0.0001) were seen in the G1 group (19.36 ± 0.49) (Figure-3b). Significant increases in the values of MCH were observed in the G6 (20.61 ± 0.34), G4 (20.72 ± 0.31), G9 (20.93 ± 0.25), G3 (21.20 ± 0.31), G5 (21.25 ± 0.25), G2 (21.43 ± 0.27), and G7 (21.62 ± 0.28) groups (Figure-3b). The highest MCH value was observed in the G8 group (21.77 ± 0.28) (Figure-3b). The lowest MCH concentration values (p < 0.039) were observed in G3 (28.93 ± 0.44) (Figure-3b). Non-significant increases in the value of mean corpuscular hemoglobin concentration was observed in G1 (29.50 ± 0.40), G8 (29.64 ± 0.33), G7 (29.78 ± 0.44), G2 (29.95 ± 0.34), G5 (30.08 ± 0.24), G4 (30.22 ± 0.24), and G9 (30.44 ± 0.23) groups, and the highest value was observed in the G7 group (30.69 ± 0.24) (Figure-3b).

The lowest values of blood platelets (p < 0.003) were seen in the G3 (452.80 × 10^3^/μL ± 16.24) and G7 (454.85 × 10^3^/μL ± 25.62) groups (Figure-3c). Non-significant increases in the values of blood platelets were observed in G9 (477.44 × 10^3^/μL ± 20.78), G5 (478.00 × 10^3^/μL ± 26.95), G8 (492.59 × 10^3^/μL ± 15.89), G2 (527.00 × 10^3^/μL ± 20.09), G4 (542.67 × 10^3^/μL ± 21.87), and G1 (557.50 × 10^3^/μL ± 30.98) groups, while the highest value was observed in the G6 (573.96 × 10^3^/μL ± 35.55) group (Figure-3c).

#### Biochemical profiles

Total proteins, albumin, globulin concentrations, and albumin/globulin ratio showed no significant difference between the non-infected control and *C. parvum-*infected groups that received or did not receive any treatments (p < 0.0001) (Table-4). Hence, *C. parvum* infection-induced systemic pathological changes likely need more time to manifest as these blood biochemical parameters. Thus, more parameters should be determined to differentiate the systemic responses to *C. parvum* infection in rats.

**Table-4:** Blood biochemical parameters and oxidative stress biomarkers determined in experimentally infected rats treated with different preparations applied during the study.

Blood parameters	Rats groups									p-value

	G1: Healthy, non-infected and non-treated	G2: Experimentally infected with 10^5^ *C. parvum* oocysts	G3: Healthy, non-infected, treated with *C. camphora* oil	G4: Healthy, non-infected rats, treated with AgNPs (50%)	G5: Healthy, non-infected rats, treated with ZnNPs (50%)	G6: *C. parvum*- infected rats treated with NTZ Reference drug	G7: *C. parvum*- infected treated with extract.	G8: *C. parvum*- infected treated with AgNPs	G9: *C. parvum*- infected rats treated with ZnNPs	
Total protein g/dL	6.76 ± 0.54	7.90 ± 0.35	7.45 ± 0.28	7.00 ± 0.40	6.90 ± 0.23	7.70 ± 0.17	7.50 ± 0.16	7.00 ± 0.40	6.90 ± 0.23	0.413
Albumin g/dL	4.25 ± 0.09^ab^	4.30 ± 0.32^ab^	4.30 ± 0.40^ab^	-	-	4.00 ± 0.12^a^	4.30 ± 0.70^ab^	4.90 ± 0.45^b^	4.90 ± 0.25^b^	0.143
Globulin g/dL	2.49 ± 0.51^a^	3.56 ± 0.41^ab^	3.50 ± 0.30 ^ab^	-	-	4.00 ± 0.40^b^	3.50 ± 0.17^ab^	3.70 ± 0.35^ab^	3.70 ± 0.20^ab^	0.110
Albumin/Globulin ratio	2.38 ± 0.52	1.50 ± 0.30	1.09 ± 0.12	-	-	1.10 ± 0.10	1.09 ± 0.06	1.00 ± 0.90	1.00 ± 0.52	0.151
AST U/mL	92.51 ± 8.53^a^	150.17 ± 3.51^c^	147.00 ± 5.00^c^	124.90 ± 3.02^b^	125.85 ± 4.22^b^	116.30 ± 6.80^b^	125.00 ± 5.20^b^	124.90 ± 3.02^b^	125.85 ± 4.22^b^	0.0001
ALT U/mL	96.93 ± 3.14^c^	53.00 ± 1.15^b^	52.5 ± 1.09^b^	46.99 ± 1.01^a^	47.09 ± 1.07^a^	47.50 ± 0.75^a^	46.90 ± 1.05^a^	46.99 ± 1.01^a^	47.09 ± 1.07^a^	0.0001
Urea g/dL	47.68 ± 10.07^a^	98.67 ± 12.93^b^	43.95 ± 1.09^a^	-	-	25.50 ± 3.81^a^	37.05 ± 2.15^a^	36.90 ± 2.04^a^	37.00 ± 1.18^a^	0.0001
Urea nitrogen g/dL	35.28 ± 10.03^ab^	55.67 ± 6.13^c^	21.02 ± 0.90^a^	-	-	49.33 ± 5.77^bc^	17.04 ± 1.08^a^	16.99 ± 0.57^a^	17.09 ± 1.18^a^	0.001
Glutathione peroxidase U/mL	35.20 ± 1.20	17.00 ± 0.60*	33.40 ± 1.20	33.40 ± 3.00	32.00 ± 2.10	22.83 ± 0.75*	35.40 ± 0.80	37.20 ± 1.50	36.10 ± 2.00	0.001
Superoxide dismutase U/mL	145.00 ± 4.00	67.80 ± 4.00*	143.00 ± 4.00	142.00 ± 6.00	147.00 ± 2.00	97.10 ± 4.50*	133.50 ± 2.00	148.00 ± 3.00	146.00 ± 3.00	0.001
Catalase U/mL	242.20 ± 8.00	175.00 ± 5.30*	239.20 ± 8.00	240.50 ± 5.00	245.00 ± 7.00	185.10 ± 9.00*	269.50 ± 4.00	255.20 ± 8.00	250.50 ± 10.00	0.001

Data expressed as means ± standard deviation. Means with different superscripts (a, b, c) within row are significant at p *<* 0.05. *C. parvum*=*Cryptosporidium parvum, C. camphora*=*Cinnamomum camphora*, AgNPs=Silver nanoparticles, ZnNPs=Zinc oxide nanoparticles, NTZ=Nitazoxanide

The results of the present study (Table-4) showed a highly significant increase in serum AST in both *C. parvum-*infected and non-infected rats. G1 had the lowest AST levels while G2 and G3 groups showed the highest AST concentrations (p < 0.0001). Rats in the G6, G3, G4, and G5 groups had lower AST levels than those in G2 but higher AST levels than those in the G1 group (p < 0.0001). In addition, there was a significant increase in serum ALT levels in G2 compared to those in the G1 group. Furthermore, the G1 group showed the highest ALT levels. In addition, the G2 and G7 groups had higher ALT concentrations than the G6, G3, G4, and G5 groups, which showed significantly lower ALT levels than those in the G1 group (p < 0.0001). Compared to non-infected and infected rats’ groups, G2 had the highest urea and urea nitrogen concentrations (p < 0.0001). Urea nitrogen reached its minimum values in G7, G8, and G9 groups (p < 0.0001).

#### Pro-inflammatory and oxidative stress biomarkers

The lowest concentrations of interferon (IFN)-γ (Figure-3c) were seen in the negative control (94.53 ± 0.21), and no significant increase (p < 0.024) was reported in *C. parvum*-*C. camphora* G7 (158.28 ± 32.74), *C. parvum*-ZnNPs G9 (167.63 ± 32.66), ZnNPs G5 (175.27 ± 72.88), *C. parvum*-AgNPs G8 (216.06 ± 63.56), AgNPs G4 (289.98 ± 156.06), and *C. parvum* positive control G2 (333.14 ± 64.64) groups. The highest levels were recorded in *C. camphora* oil G3 group (415.73 ± 99.68). IL-4 level was low (Figure-3c) in the *C. parvum* positive control group (179.24 ± 37.48), yet significantly increased (p < 0.0001) in AgNPs (369.10 ± 52.93), *C. parvum*-AgNPs (383.23 ± 46.26), *C. parvum*-*C. camphora* (408.65 ± 30.85), *C. camphora* oil (422.77 ± 19.89), and *C. parvum-*NTZ (448.20 ± 19.80), ZnNPs (504.70 ± 40.06), and negative control (521.70 ± 0.29) groups. The highest values were recorded for rats in the *C. parvum*-ZnNPs group (569.67 ± 24.64). The enzymatic activities of the antioxidants were estimated, and the readings are represented as U/mL of the enzyme (Table-4).

#### Histopathological findings

**Figure-4:**
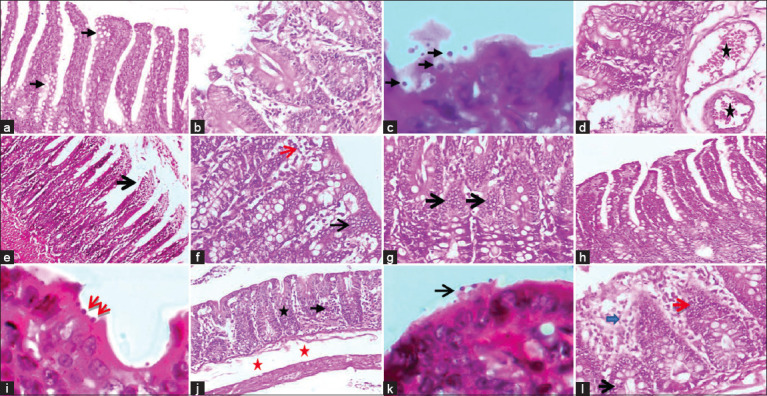
Sections in treated rats’ ileum stained with H&E showing: a) Healthy non-infected rat with the characteristic finger-like shape of villi and numerous goblet cells (black arrows, ×100). b, c and d) *C. parvum* infected rat showed degenerative and necrotic changes with sloughing upper tips of villi associated with lymphocytic cell infiltration (b; ×200), multiple aggregations and/or clusters of basophilic rounds to oval *C. parvum* oocysts attached to surface epithelium (black arrows, c; ×1000), and severe dilatation and congestion of blood vessels with subepithelial and submucosal oedema (stars, d; ×200). e). Rat treated with *C. camphora* oil extract showed necrosis and sloughing of surface epithelium of villi (black arrow, X200). f) AgNPs treated rats showed slight hyperactivity of intestinal glands (black arrow), mild subepithelial oedema and lymphocytic cells infiltration (red arrow, ×200); g) ZnNPs treated rats showed hyperactivity and hyperplasia of intestinal glands with lymphocytic cells infiltration (black arrows, ×200). h) *C. parvum* infected rat treated with Nitazoxanide showing finger–like shaped villi and numerous goblet cells (×100). i) *C. parvum* infected rat treated with *C. camphora* oil showed clusters of oval *C. parvum* oocysts attached to brush border of epithelial cells of surface mucosa (red arrows, ×1000). j) *C. parvum* infected rat treated AgNPs showed hyperactivity and hyperplasia of intestinal glands (black star), lymphocytic cells infiltration (black arrow), moderate subepithelial and submucosal, and inner circular and outer longitudinal muscles oedema (red stars). k) presence of few numbers of basophilic round to oval *C. parvum* oocysts attached to surface epithelium (black arrow, ×1000). l) *C. parvum* infected rat treated with ZnNPs showed hyperactivity and hyperplasia of intestinal glands (red arrow), mild subepithelial oedema and inflammatory cell infiltrations (blue arrow), notes of mitotic divisions of glandular epithelium (black arrow, ×100).

The histological architecture of ileum of healthy non-infected rat group (G1) showed normal structure of villi with finger–like shape and the presence of numerous goblet cells (Figure-4a). In *C. parvum* infected rats (G2): ileum revealed a shortening and blunting or widening of intestinal villi with depletion of goblet cells. Moreover, there were an atrophy, degeneration, and necrosis with sloughing of upper tips of villi (Figure-4b). Large numbers of basophilic, round to oval bodies (*C. parvum* oocysts) attached to brush border of epithelial cells in surface mucosa were observed (Figure-4c). Significant lymphocytic cells infiltration in lamina propria associated with pronounced submucosal oedema as well as dilatation and congestion of blood vessels were noticed (Figure-4d). In healthy non–infected rats treated with *C. camphora* oil extract (G3), there were degeneration and necrosis with sloughing of upper tips of villi associated with lymphocytic cell infiltration (Figure-4e).

In healthy, non-infected rats treated with AgNPs (G4), slight hyperactivity of crypts of intestinal glands (crypts of Lieberkühn) associated with lymphocytic cell infiltration and mild oedema in lamina propria and submucosa were found (Figure-4f). While healthy rats, non-infected treated with ZnNPs (G5) exhibited marked hyperplasia and hyperactivity of crypts of intestinal glands associated with lymphocytic cell infiltration (Figure-4g). In *C. parvum* infected rats treated with Nitazoxanide (G6), in almost of examined cases, there was a restoration of histological and symmetrical architecture of villi associated with decreased lymphocytic cell infiltration and absence of oocysts (Figure-4h). In few cases, sub mucosal oedema associated with congestion and dilatations of blood capillaries were observed. In *C. parvum* infected rats treated with *C. camphora* oil extract (G7), ileum revealed a shortening and blunting or widening of intestinal villi. clusters of *C. parvum* oocysts were seen attached to brush border of epithelial cells of surface mucosa with marked lymphocytic cell infiltration in the lamina propria and submucosa especially between intestinal glands. Also, dilatation and congestion of blood capillaries were noticed (Figure-4i).

In *C. parvum* infected rats treated with *C. camphora* oil-*Ulva fasciata*-AgNPs (G8), there was a significant improvement in histopathological changes. Ileum showed shortage and thickening of villi. Moreover, hyperplasia of goblet cells and hyperactivity of the intestinal glands, also subepithelial oedema associated with lymphocytic cell infiltration were noticed (Figure-4j). Few numbers of oocysts at the brush border of epithelial cells were observed (Figure-4k). Moderate submucosal and inner circular and outer longitudinal muscle oedema were also seen. In *C. parvum* infected rats treated with *C. camphora* oil-*Ulva fasciata*-ZnNPs (G9), a remarkable improvement in histopathological picture was seen. Ileum revealed shortening and widening of villi with significant decrease in the numbers of oocysts at the surface epithelium. There was a hyperactivity of intestinal glands as indicated by increase the number of mitotic divisions of glandular epithelium, in addition to hyperplasia of goblet cells. Moreover, mild inflammatory reaction in the form of oedema of lamina propria, submucosa with inflammatory cellular infiltration mainly lymphocytes and macrophages (Figure-4l).

#### Genotoxicity assessment: micronucleus frequency and chromosomal aberrations percent

**Figure-5:**
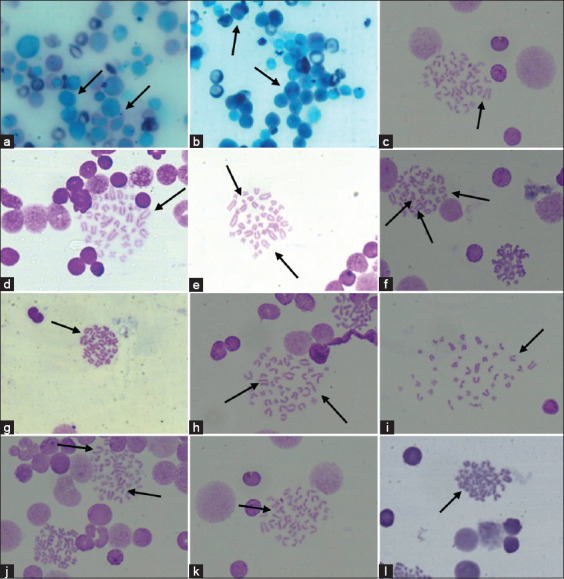
Genotoxicity assessments in treated rats groups inspected in bone marrow: a) micronucleus, b) binucleated cells, c) gap, d) break, e) break and fragment, f) break, fragment and centromeric fusion, g) centromeric fusion, h) break and Fragment, i) break, j) break and gap, k) fragment, l) fragment, all images were taken at 100 × magnification.

Table-5 presents the micronuclei and estimated scores of chromosomal aberrations (Figure-5a-l) present in the types and numbers of rats in Groups 1 to 9 (except 2). The combined data of the two-time intervals of treatments (21-and 90-days PI) revealed a significant variation among the treatments and control groups in terms of micronuclei and chromosomal aberrations (Table-5). Micronuclei and chromosomal aberrations were 7% and 6.4% after 90- and 21-days PI, respectively. In the G6 group, the highest values were recorded after 21- and 90-days PI (10.4% and 11%, respectively). The lowest values were observed in the G9 group (7% and 7.2%, respectively) after 21- and 90-days PI. The highest percentages of chromosomal aberrations after 21- and 90- days were observed in the G6 group (37.2% and 39.2%, respectively). On the other hand, the lowest percentage was recorded in the G9 group (25% and 26.2% at 21- and 90-days PI, respectively). The percentages of chromosomal aberrations in the G1 group were 22.4% and 24.2% after 21- and 90-days PI, respectively. The chromosomal aberration type with the highest frequency was that of breaks, which were recorded after 21- and 90-days PI in G6 (40% and 42%, respectively). On the other hand, the chromosomal aberration type with the lowest frequency was centromeric fusion, recorded after 21 days in G4 and G7 groups (Table-5).

**Table-5:** Frequency of micronuclei and chromosomal aberration in bone marrow cells of treated rats’ groups.

Groups	Days post- treatments	Studied cells/group	micronuclei	micronuclei %	Binucleate cells	Abnormal cells no.	Chromosomal aberration types %				Chromosomal aberration %

							Deletion and Fragments	Gaps	Breaks	Centromeric fusion	
G1: Healthy, non-infected and non-treated	21	500	32	6.4	16	112	18	10	18	18	22.4^j^
	90	500	35	7	19	121	20	10	19	18	24.2*^i^*
G3: Healthy, non-infected, and treated with *C. camphora* oil	21	500	41	8.2	14	155	20	30	29	21	31*^d^*
	90	500	43	8.6	15	166	21	32	30	25	33.2*^c^*
G4: Healthy, non-infected rats, treated with AgNPs (50%)	21	500	38	7.6	12	142	26	24	27	15	28.4^ef^
	90	500	40	8	15	152	25	25	27	20	30.4*^d^*
G5: Healthy, non-infected rats, treated with ZnNPs (50%)	21	500	40	8	17	148	22	24	25	20	29.6^de^
	90	500	42	8.4	18	150	20	25	25	20	30^de^
G6: *C. parvum*-infected rats treated with NTZ Reference drug	21	500	52	10.4	18	186	30	26	40	20	37.2^b^
	90	500	55	11	19	196	32	28	42	20	39.2^a^
G7: *C. parvum*-infected treated with extract	21	500	38	7.6	9	130	21	22	25	15	26^gh^
	90	500	39	7.8	11	137	20	22	27	18	27.4^fg^
G8: *C. parvum*-infected treated with AgNPs	21	500	50	10	9	184	23	36	35	31	36.8^b^
	90	500	45	9	9	187	25	38	36	34	37.4^b^
G9: *C. parvum*-infected rats treated with ZnNPs	21	500	35	7	6	125	20	26	20	18	25^hi^
	90	500	36	7.2	9	131	21	26	20	19	26.2^gh^

Mean value was compared using the two-way analysis of variation (ANOVA) followed by Duncan’s multiple range test (p < 0.05). Different small letters within the same line indicated significant difference among treatment at p *<* 0.05

## Discussion

Although various medicines against *C. parvum* have been tested, their efficacy has not been demonstrated. Therefore, approved treatment for cryptosporidiosis is challenging [1–3, 10, 15, 41]. In our study, bio-NPs loaded with *C. camphora* oil extract, which has been reported to exhibit beneficial pharmaceutical and biological effects [1, 28, 41, 42], were investigated against cryptosporidiosis in experimentally infected rats. The results obtained were similar to those reported previously by Chen and Dai [42] and Hyldgaard *et al*. [43], where an ethanol extract of *C. camphora* showed remarkable acaricidal activity. After a 7-day treatment in a potted seedling experiment, 2,4-di-tert-butylphenol and ethyloleate, with lethal concentration 50 values of 1850.94 and 2481.65 mg/kg, respectively [28, 42], were the most active constituents of the extract. Linalool is a major contributor to the insecticidal and repellent properties of the extracted oil [42, 43]. Moreover, our results agree with those of previous studies by Remmal *et al*. [28] and Kandale *et al*. [44], which reported the insecticidal potential of camphor essential oils against cotton aphids. Lethal concentration 50 values of 245.79, 274.99, and 146.78 mg/L were reported after 48 h of treatment for three different essential oil preparations [42, 43].

Conjugation of *C. camphora* oil extract with NPs through the marine algae *U. fasciata* facilitated their homogeneity, improved the tidiness of the particles, enabled nano-sized construction, and enhanced all pharmaceutical effects [23–25]. *U. fasciata* contains biologically active pharmaceutical compounds with various therapeutic benefits [44, 45]. Five compounds, including azelaic acid, n-pentadecanoic acid, hexa-hydro-farnesyl acetone, palmitic acid, and palmitic acid ethyl ester, have been previously identified in *U. fasciata*. Pharmacokinetically, they have non-mutagenic, noncarcinogenic, and non-toxic therapeutic effects [25, 46, 47]. Biofabricated NPs have been proposed to improve the medical effect of *C. camphora* oil extract on *C. parvum* These excellent biotic control agents are volatile or ethereal oils, comprising mixtures of odorous and volatile bioactive compounds in the form of natural complex secondary metabolites, characterized by a lower density than water, and show low toxicity to animals but high volatility and toxicity to oocysts, microbes, and pests [19, 48]. Subsequently, biofabricated multipartite essential oils-*U. fasciata*-inorganic base-NPs have gained importance as a promising class of ecological products with *in vivo* antidiarrheic and oocysticidal activities, immune-enhancing and growth-promoting effects in experimentally infected rats [27, 28]. The presence of beneficial radicals was proved from the TEM and SEM images. In addition, EDX analysis revealed 5 and 11 clear peaks within AgNPs and ZnNPs, respectively. Some values were similar between the two preparations, which could be attributed to the *C. camphora*-*U. fasciata* part of structure.

*Cinnamomum camphora* oil extract conjugated with ZnNPs showed the best therapeutic effect on *C. parvum* in experimentally infected rats, where no oocysts were found on day 17 PI, with the highest reduction percentage (100%). The conjugation of AgNPs with *C. camphora* oil also reduced the number of oocysts, as there were negligible counts of oocysts on day 18 PI with no statistically significant differences (p > 0.01) compared with rats treated with ZnNPs. On day 18 PI, AgNPs achieved a 94% reduction in oocyst count, which reached 100% after 2 days. This could be explained by the hypothesis that nano formulations help improve the uptake, bioavailability, and absorption of supplements compared to bulk equivalents [49]. These results agree with those of previously discussed nano therapy-based approaches [7, 19, 27, 47]. Therefore, it could provide a strategy for improving the effect of any *Cryptosporidium*-targeting material and achieving good antiparasitic activity [14, 16]. Many types of NPs that vary in their simplicity of preparation, non-toxic properties, stability, biodegradability, and cost efficiency have shown anticryptosporidial actions; hence, they have the ability to break the *Cryptosporidium* oocyst wall [13, 35].

Previously, NTZ therapy showed only a slight effect against diarrhea and/or enteritis caused by *Cryptosporidium* spp. [50, 51]. In the present study, NTZ caused a marked decrease in the mean oocyst count after administration. Similarly, *C. camphora* oil showed an anticryptosporidial effect similar to that of NTZ from day 14 PI, with no statistically significant difference between the NTZ-and *C. camphora* oil-treated groups. Oocyst counts in the *C. camphora* oil-treated group were significantly decreased (p > 0.01) on days 16, 17, and 18 PI compared to those in the NTZ-treated group. The anticryptosporidial effect of *C. camphora* oil might be because *C. camphora* is a terpene [43] with reported medical benefits such as anti-inflammatory, antiplasmodial, antioxidant, anticancer, digestive enhancement, and many other properties [51]. In the present study, *C. camphora* demonstrated oocysticidal activity *in vitro* and *in* *vivo* against cryptosporidiosis, as well as previously described by Remmal *et al*. [28] anticoccidial action.

Blood tests and chemistry of any animal provide an opportunity to clinically investigate the presence of different metabolites and other constituents in the circulatory system; thus, it plays a key role in the assessment of the physiological and/or pathological status of the host [13]. With regard to hematological and chemical indicators, the nano-preparations were successful not only in overcoming *C. parvum* infection in the shortest time [20] but also in ameliorating the pathological changes to levels close to those observed in the negative control group [18, 19]. Hepatic insufficiency due to cryptosporidiosis can be determined by estimating the total protein and enzymatic activities of AST and ALT [52]. In the present study, infected non-treated (positive control) rats and those treated with *C. camphora* oil extract had higher ALT concentrations than rats infected with *C*. *parvum* treated with NTZ, *C. camphora* oil, AgNPs, and ZnNPs, which showed significantly lower levels than the negative control rats (p = 0.0001). High levels of AST and ALT are characteristic of viral hepatitis, cardiac infarction, and parasitic infection. Aspartate aminotransferase catalyzes the conversion of alanine to pyruvate and glutamate and is released in a similar manner [36]. Therefore, the ALT level is a more specific and sensitive parameter for detecting liver injury. Elevated levels of both enzymes are indicative of hepatocellular necrosis, cellular leakage, and loss of functional integrity of the cell membrane in liver tissue [36, 52]. Liver parenchymal cells are responsible for the synthesis of albumin, most globulins, AST, and ALT levels. An increase in their synthesis may explain their elevation in the presence of increased biliary pressure [52, 53]. In addition, in addition to other measurements in the infected rats, elevated levels of serum urea were also observed. These results are consistent with previous reports by Finco and Duncan [53], suggesting that serum urea level is an index of renal damage and dysfunction. Therefore, diminished glomerular function rates reduce kidney filtration of urea and/or other metabolites during renal dysfunction [54, 55].

The innate immune system is considered to be the primary line of defense against pathogens and triggers the cellular response and the immune–inflammatory cascade [8, 56]. Oxidative stress, and hence parasitic infection, is accompanied by oxidative status as a modulator of immune activation [57, 58]. Oxidants are first released by immune cells that use their cytotoxic effects to kill the pathogen [59, 60]. Second, oxidants are by-products of oxygen consumption that increase metabolic activity, resulting in the generation of more toxic oxidants [61–64]. Third, degradation products of the parasite’s metabolism aggravate oxidative repercussions, mainly during provisioning when energetic demands are high [34, 65]. These findings justify the increased serum concentrations of SOD and CAT in rats treated with AgNPs and ZnNPs. Chemically, oxidative stress is associated with increased production of oxidizing species or a significant decrease in the effectiveness of antioxidant defenses, such as GPx [66, 67]. Therefore, the obtained data demonstrate the advantages of different types of treatments. Therefore, the effects of oxidative stress depend on the extent of these changes, and the cells are able to overcome small perturbations and regain their original state. However, more severe oxidative stress can cause cell death, moderate oxidation can trigger apoptosis and more intense oxidative stress may cause necrosis [36, 68]. In the present study, IFN-γ levels were significantly increased in the *C. parvum*-infected rat group compared to the non-infected control group. These results agree with those of other animal models where IFN-γ production was revealed to be important for early parasite control [32, 69]. Conversely, deficiency of IFN-γ-mediated signaling in enterocytes, macrophages, and dendritic cells may antagonize early parasite control [8, 55]. In the present study, *C. parvum*-infected rats treated with NTZ had the lowest IFN-γ concentrations compared with healthy non-infected and infected rats treated with ZnNPs and AgNPs alone. In line with our results, infected immunocompromised mice either untreated or treated with NTZ had the lowest concentrations of IFN-γ compared to immunocompromised mice treated with Melfoquine or Melfoquine and NTZ, and immunocompromised untreated negative control [13]. In the present study, both *C. parvum*-infected rats and *C. camphora* oil extract-treated rats had the highest concentrations of IFN-γ [13]. Our results also revealed that treatment with *C. camphora* oil extract, AgNPs, and ZnNPs might stimulate the production of IFN-γ in infected groups, and the strongest effect was observed with AgNPs. However, the production of IFN-γ was significantly decreased in the infected group treated with NTZ. In addition, early protection against cryptosporidiosis occurs by enhancing Th1-and Th2-mediated mucosal immune responses through immunoglobulin (Ig) G and IgE production, which inhibit parasite development. The immune response is mediated by IL-4 [9, 70]. In the present study, IL-4 concentrations increased after ZnNPs administration in infected rats. However, the highest IL-4 concentration was observed in rats infected with *C. camphora* oil extract, which is in agreement with the results reported previously by Habeeb Rahuman *et al*. [71]. Cooperation between IL-4 and IFN-γ (MASK6) has been reported in parasite killing, which may occur through intracellular Fe^2+^ deprivation [72, 73]. *Cryptosporidium* specifically invades enterocytes, and host innate resistance and parasitic clearance rely on the production of cytokines, such as IFN-γ, as well as cell-mediated immunity [74]. Our histopathological data revealed a significant increase in cellular infiltration and the skin test. These observations are supported by previous studies which illustrated that cryptosporidiosis was accompanied by the production of pro-inflammatory cytokines and chemotaxis, which lead to natural killer cell, lymphocyte, and macrophage infiltration [6, 75, 76].

Intestinal epithelial architecture includes enterocytes, Paneth, goblet, and tuft cells which play various roles in immune defense and mucosal homeostasis. A subset of enteric pathogens is restricted to the epithelial layer, where their interactions with enterocytes are likely to be key determinants of illness [77]. In our study, we observed shortening and blunting of intestinal villi with depletion of goblet cells, atrophy, degeneration, and necrosis with sloughing of the upper tips of the villi with large numbers of basophilic, round to oval bodies (*C. parvum* oocysts) attached to the brush border of epithelial cells in the surface mucosa. Similar results were obtained in experimentally infected mice, which showed loss of ileocecal brush borders and villous architecture, with marked villous atrophy, shortening, and broadening, with the presence of round to oval, purple *Cryptosporidium* oocysts in the intestinal lumen [2, 3, 15, 78]. In addition, villus height was significantly reduced when the number of *C. parvum* oocysts was maximal [79]. In addition to the presence of edema, dilated congested blood vessels and mild inflammatory cell infiltration were recorded in experimentally infested mice treated with either mefloquine or NTZ [13]. Infected rats treated with AgNPs showed significant improvement with shortened and thickened intestinal villi, scanty numbers of oocysts, mild edema, and mild lymphocytic infiltration. In our study, rats treated with AgNPs and ZnNPs showed remarkable improvement, with shortening and widening of villi associated with hyperactivity of intestinal glands (crypts of Lieberkühn), hyperplasia of goblet cells, and a mild inflammatory reaction in the form of edema of the lamina propria and submucosa with inflammatory cellular infiltration of mainly lymphocytes and macrophages, which were also observed in healthy rats treated with only the NPs.

The genotoxic effects of ZnNPs and AgNPs multipartite particles in the bone marrow of treated rats with ZnNPs and AgNPs showed similar significant improvement. The results were confirmed by the analysis of the combined data of two treatment time intervals (21-days and 90-days PI), which revealed significant differences between the treatment and control groups. These observations agree with previous reports on the role of *U. fasciata* primary and secondary metabolites and potential therapeutic compounds that adversely affect apoptotic and mutagenic cascades within infected host cells [23–27]. On the other hand, essential oils are substances with various characteristics that can cause mutations, genotoxicity, and carcinogenicity in mammals; hence, the effect of essential oils on non-target organisms should also be identified [7, 80]. This could justify the recorded scores of micronuclei and chromosomal aberrations in rats treated with *C. camphora* oil extract; hence, some of its constituents still require safety risk assessment. The maximum daily therapeutic dose of d-camphor is about 1.43 mg/kg. This dose is relatively safe; however, long-term data are lacking. In addition, although safrole has significant anti-parasitic activity, it is also carcinogenic. In addition, linalool harms aquatic environments [8, 9]. In addition, 1,8-cineol is toxic to the respiratory and nervous systems [8, 9]. Therefore, the recorded genetic toxicity, micronucleus, and chromosomal aberrations, in addition to oxidative stress records, reflect an imbalance between the systemic manifestation of reactive oxygen species and a biological system’s ability to readily detoxify reactive intermediates and/or repair the resulting damage [39, 78]. Nonetheless, disturbances in the normal redox state of host cells can cause toxic effects through the production of peroxides and free radicals that damage all cellular components, including proteins, lipids, and DNA [39, 79].

## Conclusion

*C. camphora*, *U. fasciata*, and ZnNPs exhibited superior antiparasitic effects in rats infected with *C. parvum*, which beneficially improved the uptake, bioavailability, and absorption of supplements compared to bulk equivalents. Moreover, their effects started earlier compared with *C. camphora* oil, *U. fasciata-*AgNPs, and NTZ. In addition, both *C. camphora* extract oil and NTZ had the same antiparasitic effects against *C. parvum*. It is possible that herbal extracts are sufficient alternatives to traditional chemical drugs, especially in individuals with immunological reactions against commercial drugs. The green processes used in the biogenesis of NPs seem to be successful, simple, non-toxic, stable, biodegradable, in ecological control of many parasites, and promising applications, which demonstrate a simple, reliable, cost effective, and environmentally friendly alternative to chemical methods. Nanotechnology science is a sustainable sector.

### Data Availability

The supplementary data can be available from the corresponding author on a reasonable request.

## Authors’ Contributions

NATA: Conception and design of the study, interpretation of data, instructions of experimental infection, PM after rats’ scarification, immunological studies, and drafted and revised the manuscript. RAE and MSA: Research conception, NPs synthesis and characterization, and revised the manuscript. DS and NMFH: Experimental infection, rats monitoring, new preparations administration, PM after rats’ scarification, participated in immunological studies, and reviewed the manuscript. MEA: Hematological and biochemistry analysis, participated in administration of preparations, monitoring infected rats, PM after scarification, and reviewed the manuscript. MEHAE and MAI: Genotoxicity characterization and analysis, provided some necessary tools and chemicals, and reviewed the manuscript. KNAM and DA: Provided parasite oocysts, shared in experimental infection, parasitological examination, statistical analysis and data interpretation, and reviewed the manuscript. AMAE: Statistical analysis, interpretation of data, and drafted and revised the manuscript. HAAT: Plant biochemistry and oil characterization and provided some necessary chemicals. ASH: Immune-reactivity, biosafety, and toxicity analysis, and reviewed the manuscript. HMD: Histopathology and reviewed the manuscript. All authors have read, reviewed, and approved the final manuscript.
